# Active learning of neuron morphology for accurate automated tracing of neurites

**DOI:** 10.3389/fnana.2014.00037

**Published:** 2014-05-19

**Authors:** Rohan Gala, Julio Chapeton, Jayant Jitesh, Chintan Bhavsar, Armen Stepanyants

**Affiliations:** Department of Physics and Center for Interdisciplinary Research on Complex Systems, Northeastern UniversityBoston, MA, USA

**Keywords:** NCTracer, neural circuit tracer, machine learning, active learning, automated reconstruction, automated tracing, axon, dendrites

## Abstract

Automating the process of neurite tracing from light microscopy stacks of images is essential for large-scale or high-throughput quantitative studies of neural circuits. While the general layout of labeled neurites can be captured by many automated tracing algorithms, it is often not possible to differentiate reliably between the processes belonging to different cells. The reason is that some neurites in the stack may appear broken due to imperfect labeling, while others may appear fused due to the limited resolution of optical microscopy. Trained neuroanatomists routinely resolve such topological ambiguities during manual tracing tasks by combining information about distances between branches, branch orientations, intensities, calibers, tortuosities, colors, as well as the presence of spines or boutons. Likewise, to evaluate different topological scenarios automatically, we developed a machine learning approach that combines many of the above mentioned features. A specifically designed confidence measure was used to actively train the algorithm during user-assisted tracing procedure. Active learning significantly reduces the training time and makes it possible to obtain less than 1% generalization error rates by providing few training examples. To evaluate the overall performance of the algorithm a number of image stacks were reconstructed automatically, as well as manually by several trained users, making it possible to compare the automated traces to the baseline inter-user variability. Several geometrical and topological features of the traces were selected for the comparisons. These features include the total trace length, the total numbers of branch and terminal points, the affinity of corresponding traces, and the distances between corresponding branch and terminal points. Our results show that when the density of labeled neurites is sufficiently low, automated traces are not significantly different from manual reconstructions obtained by trained users.

## Introduction

Our understanding of brain functions is hindered by the lack of detailed knowledge of synaptic connectivity in the underlying neural network. With current technology it is possible to sparsely label specific populations of neurons *in vivo* and image their processes with high-throughput optical microscopy (see e.g., Stettler et al., 2002; Trachtenberg et al., 2002; De Paola et al., 2006; Wickersham et al., 2007; Lichtman et al., 2008; Wilt et al., 2009; Ragan et al., 2012). Imaging can be done *in vivo* for circuit development or plasticity studies (Trachtenberg et al., 2002), or *ex vivo* for circuit mapping projects (Lu et al., 2009). In the latter case, an unprecedented resolution can be achieved by first clarifying the tissue (Hama et al., 2011; Chung et al., 2013), and then imaging the entire brain from thousands of optical sections (Ragan et al., 2012). The overwhelming obstacle remaining on the way to brain mapping is accurate, high-throughput tracing of neurons (Sporns et al., 2005; Lichtman et al., 2008; Miller, 2010; Gillette et al., 2011b; Kozloski, 2011; Lichtman and Denk, 2011; Liu, 2011; Svoboda, 2011; Helmstaedter and Mitra, 2012; Van Essen and Ugurbil, 2012; Perkel, 2013). Presently, accurate traces of complex neuron morphologies can only be obtained manually, which is extremely time consuming (Stepanyants et al., 2004, 2008; Shepherd et al., 2005), and thus impractical for large reconstruction projects.

**Figure 1:**
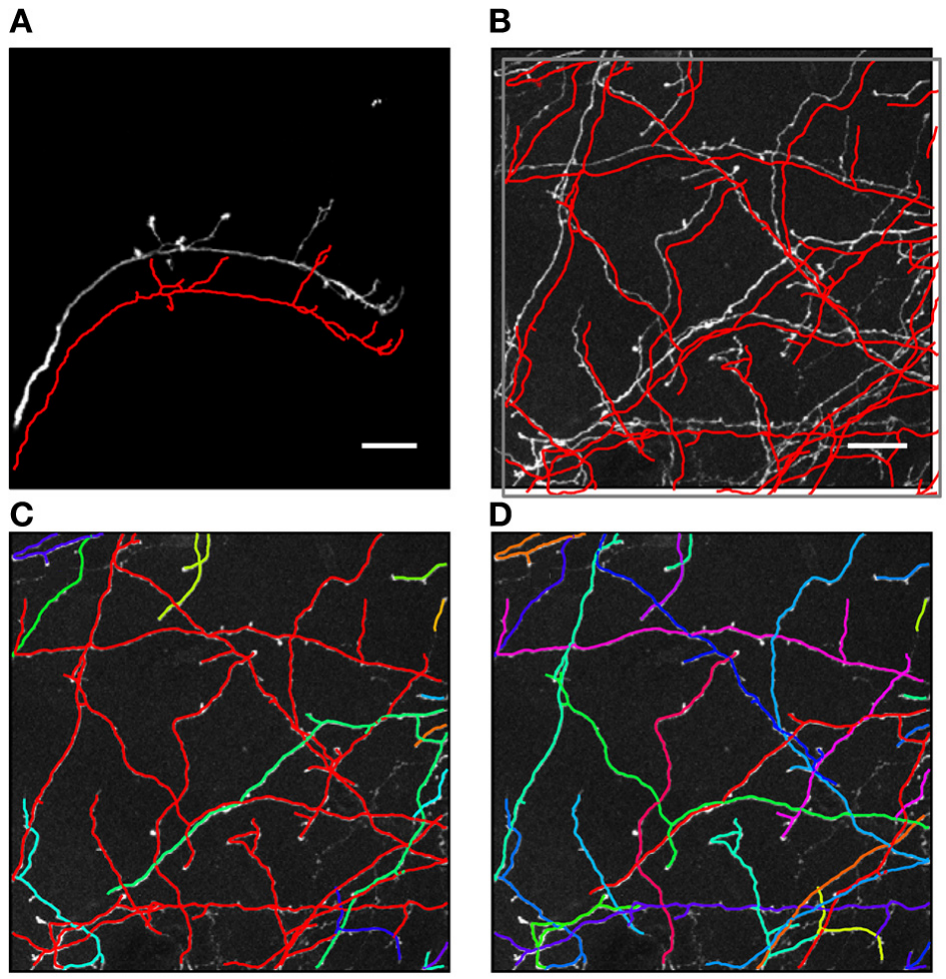
**Automated traces of neurites may contain hidden topological errors. (A)** Example of an automated trace of drosophila olfactory neuron axon (Jefferis et al., 2007). For better visibility the initial trace (red line) is shifted down with respect to the maximum intensity projection of the image stack. Due to the simple morphological structure of the neuron, as well as high and uniform contrast of the image, the initial trace accurately captures both the geometrical and topological structure of the arbor. **(B)** Maximum intensity projection of an image stack containing mouse neocortical axons of layer 6 neurons (De Paola et al., 2006). The initial trace (red lines) accurately captures the geometry of the neurites, but contains topological errors. **(C)** These topological errors are revealed after labeling each tree of the initial trace with a different color. The red tree in the image is composed of multiple axons which were erroneously connected to each other. **(D)** To correct such topological errors the initial trace is taken apart up to a level of branches and subsequently put together by using the knowledge of neuron morphology. An active machine learning framework is used to accomplish this task. Scale bars in **(A)** and **(B)** are 20 μm.

Many automated tracing algorithms have been developed in recent years [see e.g., Al-Kofahi et al., 2002; Schmitt et al., 2004; Zhang et al., 2007; Al-Kofahi et al., 2008; Losavio et al., 2008; Peng et al., 2010; Srinivasan et al., 2010; Bas and Erdogmus, 2011; Peng et al., 2011; Turetken et al., 2011; Wang et al., 2011; Xie et al., 2011; Bas et al., 2012; Choromanska et al., 2012; Turetken et al., 2012 and Meijering, 2010; Donohue and Ascoli, 2011; Parekh and Ascoli, 2013 for review]. In general, existing algorithms can accurately capture the geometrical layout of neurites but are not guaranteed to recover their correct branching topology (Figure 1). Topological errors are inevitably present in traces obtained from low signal-to-noise images, images of non-uniformly labeled neurites, or images with high density of labeled structures. Close examination of such traces often reveals topological errors such as broken branches, missing branches, and incorrectly resolved branch crossover regions (stolen branches). This is a particular concern for high-throughput projects where topological errors can accumulate over multiple stacks. For example, while tracing a long-range axon from one optical section to the next, even a very low error-rate, say 5% per section, will almost certainly lead to erroneous connectivity after about 20 sections (typically about 10 mm), rendering the trace unusable for brain mapping projects. Clearly, the rate of topological errors in automated reconstruction projects must be carefully controlled (Chothani et al., 2011).

In this study we describe an active machine learning approach (Settles, 2012) that has the potential to significantly reduce the number of topological errors in automated traces. Our algorithm first detects a geometrically accurate trace with the Fast Marching method (Cohen et al., 1994; Cohen and Kimmel, 1997; Sethian, 1999; Mukherjee and Stepanyants, 2012), which was extended to incorporate multiple seed points. Next, the initial trace is dismantled to the level of individual branches, and active learning is applied to reconnect this trace based on knowledge of neuron morphology. We show that active learning does not require large sets of training examples, and the results generalize well on image stacks acquired under similar experimental conditions. What is more, when the density of labeled neurites is sufficiently low, automated traces are not significantly different from reconstructions produced manually by trained users.

## Methods

Results of this study are based on the analyses of two datasets featured at the DIADEM challenge (Brown et al., 2011). The OP dataset includes 9 image stacks containing axons of single olfactory projection neurons from Drosophila (Jefferis et al., 2007), and the L6 dataset consists of 6 image stacks containing axons of multiple layer 6 neurons imaged in layer 1 of mouse visual cortex (De Paola et al., 2006). The NCTracer software (www.neurogeometry.net) was used to trace each image stack automatically, as well as manually. The manual traces were generated independently for each stack by three trained users.

### The initial trace of neurites

**Figure 2:**
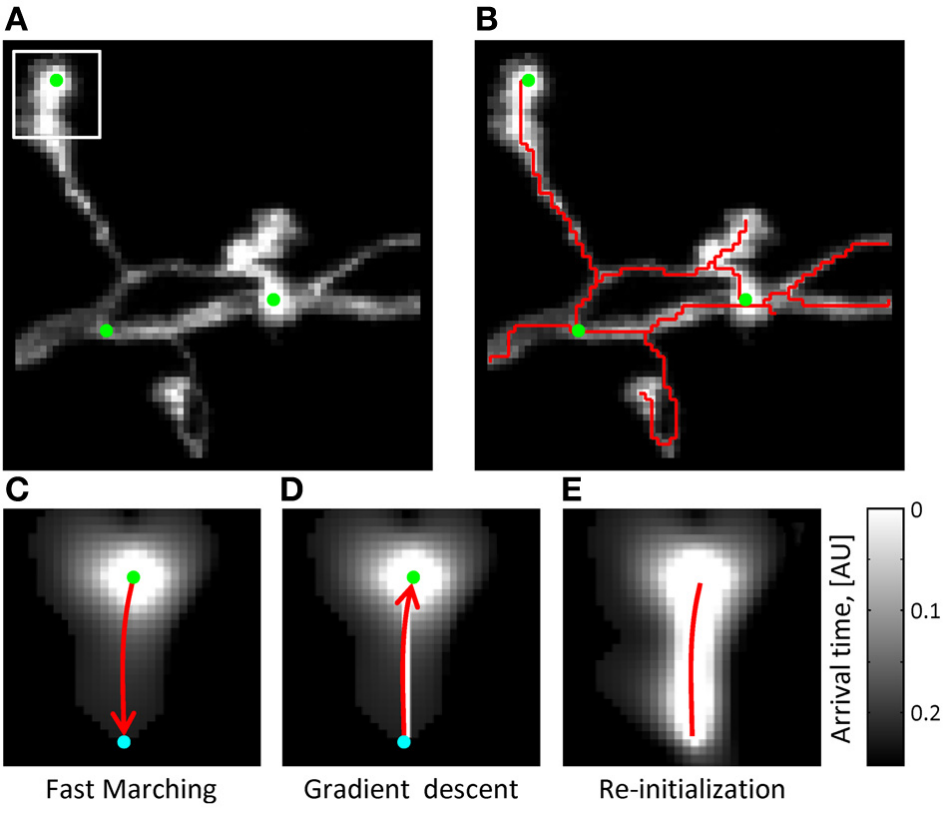
**The initial trace can be based on the solution of the Eikonal equation. (A)** Maximum intensity projection of an OP image stack. Green dots mark the locations of seed points used to initialize the Fast Marching algorithm (*T* = 0 points). The white square outlines the image section used in **(C–E)** to illustrate the main steps of the algorithm. **(B)** The initial trace resulting from Fast Marching is shown with a red line. **(C)** Arrival time map for the front originating at *T* = 0 from the location marked by the green seed point. Front propagation stops when the front reaches a user-defined Euclidean distance (cyan point). Red arrow shows the path of shortest time delay connecting the two points. **(D)** This path is obtained with the gradient descent algorithm. **(E)** The arrival time map along the path of shortest time delay is re-initialized to *T* = 0 (red line), and the Fast Marching algorithm is restarted. Note that brighter intensities correspond to smaller arrival times.

We refer to any trace providing geometrically accurate information about the layout of neurites within an image stack as an initial trace. Numerous segmentation and tracking-based methods (see e.g., Al-Kofahi et al., 2002; Schmitt et al., 2004; Zhang et al., 2007; Al-Kofahi et al., 2008; Losavio et al., 2008; Peng et al., 2010; Srinivasan et al., 2010; Bas and Erdogmus, 2011; Peng et al., 2011; Turetken et al., 2011; Wang et al., 2011; Xie et al., 2011; Bas et al., 2012; Choromanska et al., 2012; Turetken et al., 2012) can be used to produce initial traces. In this study we adapt the Fast Marching method (Cohen et al., 1994; Cohen and Kimmel, 1997; Sethian, 1999; Mukherjee and Stepanyants, 2012) to grow the initial trace from multiple seed points (Figure 2), analogous to the way light from multiple point sources spreads through a non-uniform medium. This process is described by the Eikonal boundary value problem (Sethian, 1999):

(1) $$\begin{array}{l} \left|\nabla T\left(r\right)\right|I\left(r\right)=1\\ T\left(\partial S\right)=0 \end{array}$$

In this expression, vector ***r*** represents a position in the image stack (or non-uniform medium), *I* is the image intensity normalized to the 0–1 range (analog of the speed of light in the medium), and **∇** denotes the gradient operator. Light rays originate from the boundary, ∂*S*, at time zero, and the time map, *T*(***r***), provides information about the shortest time of arrival of these rays to various locations in the image. Because higher image intensities correspond to faster speeds of light propagation, the arrival time front in the image will preferentially spread along the high intensity structures of neurites (see Figure 2C).

The Fast Marching algorithm of Sethian (Sethian, 1999) is an efficient numerical scheme for solving the Eikonal boundary value problem, Equation (1). Since the speed function in our problem is defined by the image intensity, it is always positive. For positive speed functions it is known that the Eikonal boundary value problem can be solved more efficiently than the commonly used alternative—the Hamilton-Jacobi problem of the Level Set method (Sethian, 1999). One reason is that the stability condition required for a numerical solution of the time-dependent Level Set equation is more stringent than that used to solve the Eikonal problem. Specifically, this condition requires very small time steps and thus the Level Set method is expected to be more time consuming. The second advantage of Fast Marching has to do with the outward only propagation of the fronts, which can be used to find new front points very efficiently (Sethian, 1999).

We implement the Fast Marching algorithm (Sethian, 1999) on a discrete lattice defined by the centers of image voxels, ***l*** = (*i, j, k*)^*T*^. Here the time map is evolved from the boundary at *T* = 0 by taking the upwind solution of a discretized version of Equation (1):

(2) $$[\begin{array}{l} \frac{1}{s_{x}^{2}}{\left(\max\left(T(i,j,k)-T(i-1,j,k),0\right)\right)}^{2}+\\ \frac{1}{s_{x}^{2}}{\left(\max\left(T(i,j,k)-T(i+1,j,k),0\right)\right)}^{2}+\\ \frac{1}{s_{y}^{2}}{\left(\max\left(T(i,j,k)-T(i,j-1,k),0\right)\right)}^{2}+\\ \frac{1}{s_{y}^{2}}{\left(\max\left(T(i,j,k)-T(i,j+1,k),0\right)\right)}^{2}+\\ \frac{1}{s_{z}^{2}}{\left(\max\left(T(i,j,k)-T(i,j,k-1),0\right)\right)}^{2}+\\ \frac{1}{s_{z}^{2}}{\left(\max\left(T(i,j,k)-T(i,j,k+1),0\right)\right)}^{2} \end{array}]\;I{\left(i,j,k\right)}^{2}=1$$

Parameters (*s*_*x*_, *s*_*y*_, *s*_*z*_) in this expression denote the voxel dimensions which may not be the same due to a typically lower *z*-resolution in confocal and two-photon microscopy images.

The arrival time front is initialized with *T* = 0 at multiple seed points, which are automatically generated along the structure of neurites based on image intensity (Figure 2A). As was previously described (Mukherjee and Stepanyants, 2012), the arrival time front is allowed to travel a specified distance, *D*_*max*_, to establish a local time map. The value of *D*_*max*_ has to be chosen based on two considerations: *D*_*max*_ has to be larger than the caliber of neurites (3–5*s*_*x*_ for OP and L6) not to produce short spurious branches and, at the same time, not much larger than the shortest branch that needs to be resolved by the algorithm (10*s*_*x*_ in this study). *D*_*max*_ = 15*s*_*x*_ was used throughout this study. The path connecting the farthest point of the front to the *T* = 0 boundary is then found by performing gradient descent on *T*(*i, j, k*) (see Figures 2C–E). Next, the gradient descent path is added to the boundary ∂*S* and the Fast Marching algorithm is re-initialized from the new boundary. This process continues until a stopping condition is reached, at which point the final ∂*S* defines the initial trace. The stopping condition used in this study is based on the average intensity of the last added branch. When this intensity falls below a set threshold (typically 20% of the average intensity of the existing trace), Fast Marching is paused and can then be continued or terminated by the user.

As long as the seed points used to initialize Fast Marching are located in the foreground and are connected by higher than background intensity paths, their Fast Marching fronts are guaranteed to collide. The gradient descent algorithm is invoked in this case as well. Here, gradient descent paths originating from the collision voxel back-propagate into every colliding region, thus connecting their *T* = 0 boundaries. If there is a break in intensity along a neurite linking two seed points, the Fast Marching algorithm may terminate before the fronts have a chance to collide. In addition, high levels of background intensity may lead to erroneous front collisions. These and other topological errors in the initial trace will be corrected as described in the following sections.

### Optimization of the initial trace

We represent the initial trace as a graph structure consisting of nodes linked by straight line segments. Each node, *k*, is described by its position in the stack, ***r***^*k*^ = (*x*^*k*^, *y*^*k*^, *z*^*k*^)^*T*^, and the caliber, *R*^*k*^, of the neurite at that location. Information about connectivity among the nodes is stored in the adjacency matrix, ***A***. We find this representation to be more convenient than the traditional SWC format of neuron morphology (Cannon et al., 1998) because the latter cannot be used to describe structures containing loops.

Because the initial trace lies sufficiently close to the centerline of neurites, this trace can be optimized by monitoring its fitness in response to small changes in the position and caliber of every node. The fitness function used in this study, 
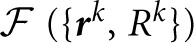
, consists of the intensity integrated along the trace and regularizing constraints on the positions and calibers of the connected nodes:

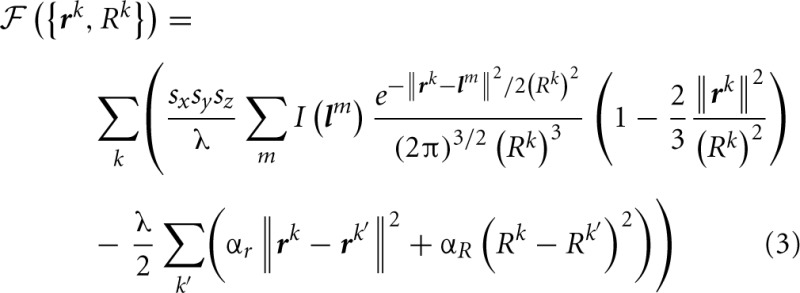



Vectors ***r***^*k*^ in this expression specify the positions of the trace vertices, while vectors ***l***^*m*^ denote the positions of voxel centers in the image stack. Index *k*' enumerates the neighbors of vertex *k*. Parameter λ denotes the average density of nodes in the trace, i.e. the number of nodes per voxel. Lagrange multipliers α_*r*_ > 0 and α_*R*_ > 0 control the stiffness of the regularizing constraints. The first term in this expression is the convolution of the image with the Laplacian of Gaussian. This convolution can be performed by using the Fast Fourier Transform (Press, 2007) or, in case of relatively small density of trace nodes, it may be faster to perform explicit summation over the index *m*. In this case, due to the fast decay of the Gaussian factor, the summation can be restricted to a small number of voxels in the vicinity of the trace (see Chothani et al., 2011 for details).

Maximization of the fitness function, 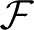, is performed with Newton's method (Press, 2007):

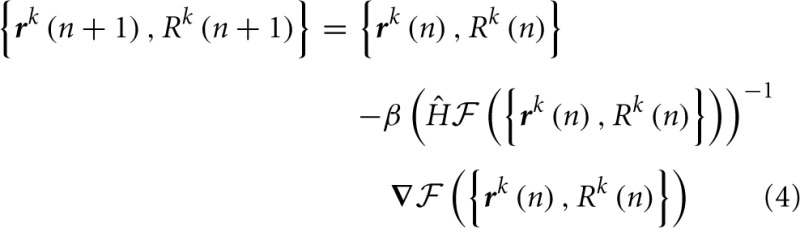



Variable *n* in this expression enumerates the iteration steps of the algorithm, parameter β > 0 controls the step size, *Ĥ* denotes the Hessian operator acting on all the node variables {***r***^*k*^(*n*), *R^*k*^*(*n*)}, and -1 in the exponent denotes matrix inversion. The positions and calibers of all nodes of the trace, including branch and terminal points, are synchronously updated at every iteration step. The values of all three terms in the fitness function are monitored during optimization. Optimization is terminated once the relative changes in all three quantities fall below 10^−8^. For the OP and L6 datasets considered in this study, the optimization procedure typically converges to the optimum solution in less than 50 steps. Optimization improves the layout of branches as well as the placement of branch and terminal points in the initial trace (Vasilkoski and Stepanyants, 2009; Chothani et al., 2011). The values of parameters α_*r*_, α_*R*_, and β are constrained by the considerations of algorithm stability, speed of convergence, and accurate representation of neurites' curvature and caliber. Some of these issues were discussed in Vasilkoski and Stepanyants (2009) and Chothani et al. (2011).

### Learning branching morphology of neurites

**Figure 3:**
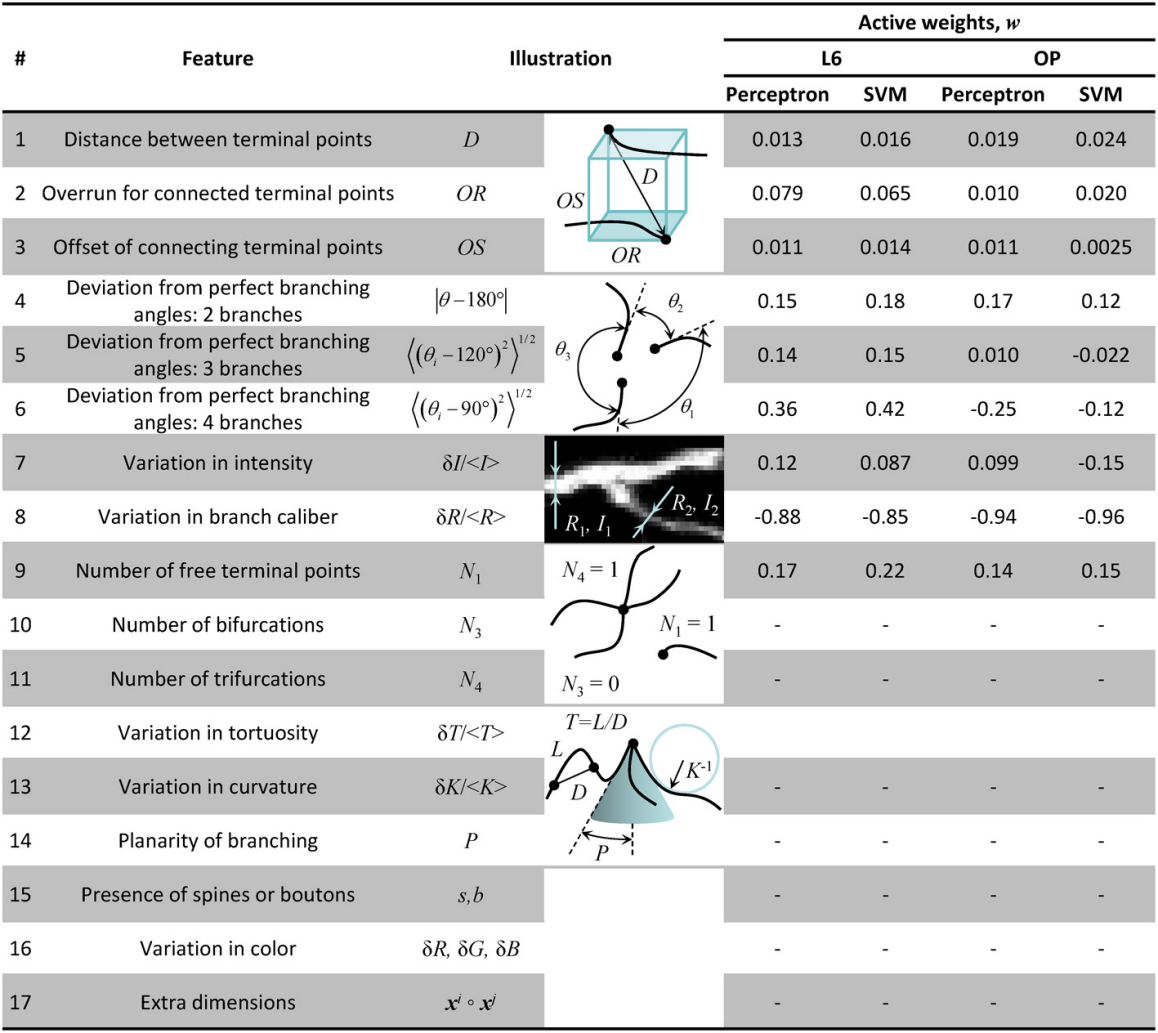
**Table of morphological features that may be useful for classification of branch merging scenarios**. Features 1–9 were used to generate the results of this study. The weights of Perceptron and SVM classifiers, obtained as a result of active training on 100 clusters of branch points, show high degree of correlation. As expected, within-dataset correlations were higher than between-dataset correlations indicative of the fact that the classifier algorithms are able to learn details of neuron morphology that are dataset-specific.

As shown in Figure 1, even when the initial trace accurately describes the geometry of neurites, it often fails to capture the correct branching topology. To address this problem, we disconnect branches of the initial trace from one another and then assemble them into tree-like structures based on prior knowledge of neuron morphology. In order to discriminate between correct and erroneous ways to assemble branches, different branch merging scenarios are evaluated in a machine learning approach by combining information about various features of the trace. Such features may include distances between branches, branch orientations, average intensities, intensity variations, branch thicknesses, curvatures, tortuosities, colors, and presence of spines or boutons. Features 1–9 of Figure 3 were used to produce the results of this study. These features were selected based on our knowledge of neuroanatomy and intuition gained from manual neuron tracing. We carefully examined the decisions we make when faced with branch merging tasks and initially created a list of 17 features that are shown in Figure 3. Features 15 and 16 are not applicable for the OP and L6 datasets as these datasets include grayscale images of axons only. Features 10–14 and 17 were tested but did not improve the performance of the classifiers. This is why the above features (10–17) were left out of the analysis. This is not to say that features 10–17 are not important; they may be useful for other dataset types.

To evaluate different branch merging patterns in the disconnected initial trace we cluster branch terminal points on the basis of their relative distances. For this, we first create an all-to-all connected graph in which nodes represent the branch terminal points. Next, the links between distant nodes (>10*s*_*x*_) are removed, exposing the clusters of nearby branch points. The threshold distance of 10*s*_*x*_ was chosen based on two considerations. First, this distance has to be larger than the voxel size (*s*_*x*_) and the size of a typical gap in intensity resulting from imperfect labeling of branches (0 for OP and ~5*s*_*x*_ for L6). Second the threshold distance has to be smaller than the typical branch length (20*s*_*x*_-50*s*_*x*_ for OP and L6). Results of branch merging are not sensitive to the precise value of this parameter in the 5*s*_*x*_–15*s*_*x*_ range. Branch merging is examined within each cluster of branch terminal points independently.

**Figure 4:**
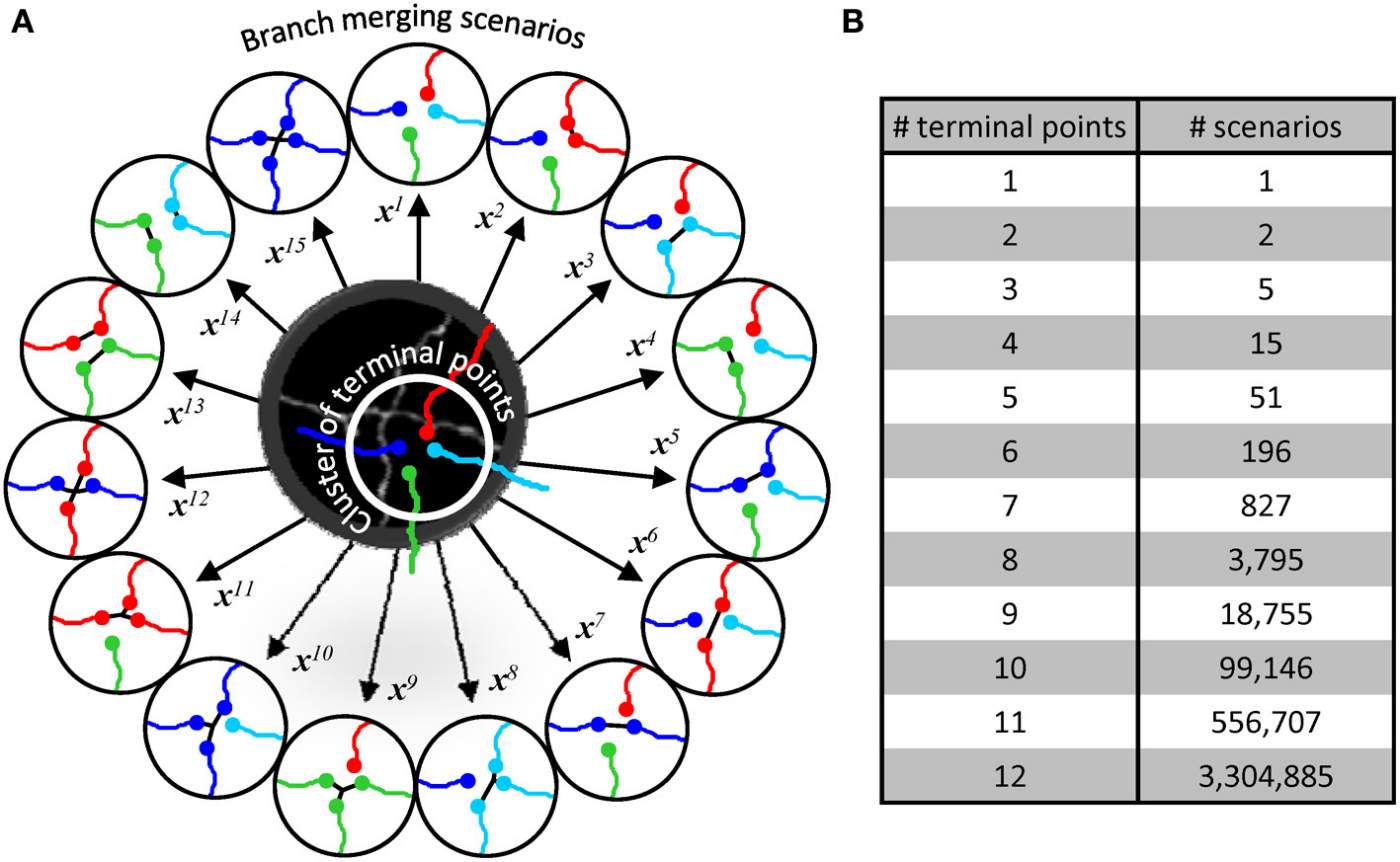
**Branch merging scenarios. (A)** Illustration in the middle shows the maximum projection image of two neurites from Figure 1B. The neurites appear fused in 3D, leading to a provisional branch point which must be resolved automatically. The initial trace of these neurites (lines are shifted down and to the right for clarity) has been disconnected at the branch point resulting in a terminal point cluster which has to be merged according to one of the 15 possible scenarios (circled traces). Each scenario, *i*, is described by a unique multi-dimensional feature vector, **x**^*i*^. **(B)** The number of possible branch merging scenarios increases exponentially with the number of terminal points, leading to large numbers of classification examples.

Within a given cluster, all possible branch merging scenarios are considered (Figure 4A), and the correct merging pattern is determined in a classification framework. Clusters containing 2 terminal points lead to two scenarios, i.e., to connect or not to connect the terminal points. Three terminal point clusters result in 5 scenarios, 4 terminal point clusters lead to 15 (Figure 4A), and the number of scenarios increases exponentially with the complexity of clusters (Figure 4B). This exponential increase gives a unique advantage to our classification approach to branch merging.

Generally, machine learning applications require large sets of labeled data. Creating such sets can be very time-consuming and, in many cases, impractical. Our training strategy circumvents this problem by exploiting the large numbers of branch merging scenarios. Labeling the correct branch merging scenario in a single cluster can provide thousands of training examples. Hence, it becomes possible to train the classifier in real time and obtain accurate results by labeling only 10–100 clusters of branch terminal points.

All possible branch merging scenarios are evaluated within a given cluster of branch terminal points. Each scenario, *i*, is characterized by a feature vector ***x***^*i*^ (Figure 4A) whose components consist of features of the trace that may be important for selecting the correct branch merging scenario (Figure 3). The problem is thus reduced to learning the best set of weights, ***w***, for discriminating between the correct and erroneous scenarios within every cluster,

(5) $$w^{T}x^{all\;erroneous\;mergers}>w^{T}x^{correct\;merger}$$

This formulation leads to another important advantage for the implementation of the classification strategy. Due to the linearity of the problem, Equation (5) can be rewritten as,

(6) $$w^{T}\left(x^{all\;erroneous\;mergers}-x^{correct\;merger}\right)>0,$$

resulting in a subtractive normalization of the feature vectors within individual clusters. Because branch merging scenarios are only compared within clusters, Equation (6) effectively normalizes for the variations in image intensity and density of neurites across clusters.

**Figure 5:**
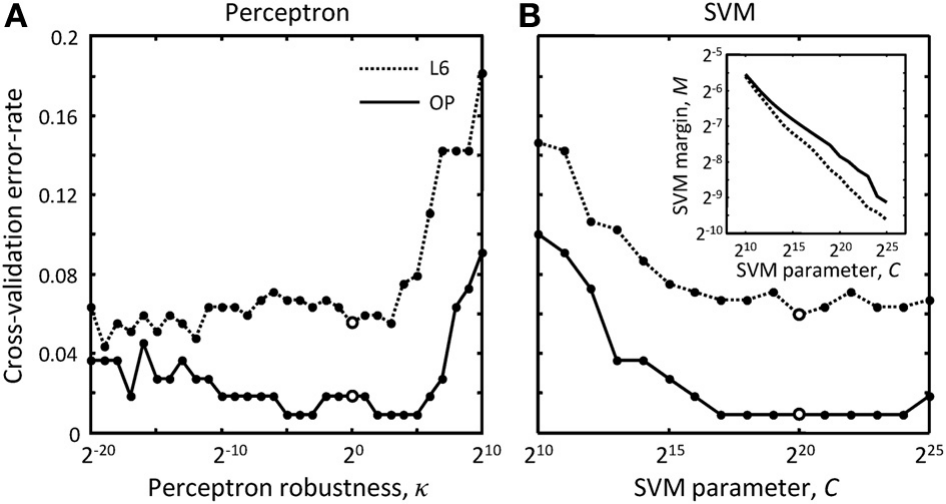
**How to choose best classification parameters. (A)** Leave-one-out cross-validation error-rate as function of the perceptron robustness parameter, κ [see Equation (7)]. **(B)** Same error-rate as function of the SVM parameter, *C* [see Equation (9)]. The inset shows how SVM margin, *M*, depends on *C*. Solid and dotted lines show the results for the OP and L6 datasets respectively. Large empty circles indicate the parameter values that were used throughout this study, κ = 2^0^ and *C* = 2^20^.

The classification problem of Equation (6) is solved with sign-constrained perceptron (Engel and Broeck, 2001) or SVM classifiers (Wang, 2005), which were modified to be able to account for the relative importance of some training examples. The sign-constrained perceptron algorithm was previously described in Chapeton et al. (2012):

(7) $$\begin{array}{l} \frac{1}{N}w^{T}\Delta x^{\mu }>\frac{\kappa }{\sqrt{N}},\;\mu =1,2,...,m\\ w_{k}g_{k}\geq 0,\;k=1,2,...,N \end{array},$$

where ***w*** is the weight vector of the perceptron classifier, Δ***x***^μ^ is the difference between the feature vectors for the erroneous merger μ and the correct merger from the same cluster, *N* is the number of features (9 features were used in this study), and *m* is the number of comparisons made (total number of scenarios minus number of clusters). The value of the parameter *g_k_* can be set to −1 or 1, constraining the corresponding weight, *w_k_*, to be negative or positive, or set to 0, in which case the weight is unconstrained. Because larger distances, overruns, and offsets of terminal points (see Figure 3) decrease the likelihood that branches should be merged, the weights of these features were constrained to be positive. In addition, the weight associated with the number of free terminal points was constrained to be positive to promote branch merging. All other weights were left unconstrained as we did not have clear motivation for doing otherwise. Hence, ***g*** = (1,1,1,0,0,0,0,0,1)^*T*^ was used in this study. Parameter κ is referred to as the perceptron robustness (analogous to SVM margin). Increasing κ should initially improve the generalization ability of the perceptron, but as the perceptron fails to correctly classify a progressively increasing number of training examples, this generalization ability should decrease. We used the leave-one-out cross-validation scheme to examine this trend. In this scheme, training is done on all but one labeled example, and the remaining example is used for validation. In Figure 5 each branch merging cluster was used once for validation and the results were averaged. Figure 5A shows that there is a large range of κ for which the perceptron performs reasonably well for both L6 and OP datasets. The value of κ was set to 1 throughout this study.

The sign-constrained perceptron problem of Equation (7) was solved by using a modified perceptron learning rule (Engel and Broeck, 2001):

(8) $$\begin{array}{l} \Delta w=\theta \left(\frac{\kappa }{\sqrt{N}}-\frac{1}{N}w^{T}\Delta x^{\mu }\right)\frac{1}{\sqrt{N}}\Delta x^{\mu }\;\\ w_{k}=w_{k}\theta \left(w_{k}g_{k}\right),\;k=1,2,...,N \end{array}$$

In this expression, Δ***w*** denotes the change in the perceptron weight vector in response to presentation of the training example μ; θ is the Heaviside step function, which is defined to be 1 for non-negative arguments and zero otherwise. The step functions in Equation (8) ensure that training is not done on learned examples, and that the perceptron weights violating the sign-constraints are set to zero at every step of the algorithm. Perceptron weights are updated asynchronously by training on examples, μ, that are drawn from the set of all examples with probabilities proportional to user-defined cluster weights, *Q*_μ_. All cluster weights are initially set to 1 and can be modified by the user to increase the probabilities with which examples from some clusters come up for training. This makes it possible to enforce learning of certain rare branch merging topologies. Though user-defined cluster weights may be used to improve the outcome of training, this feature was not examined in the present study to avoid subjectivity associated with different choices of *Q*_μ_.

An SVM classifier can also be used to solve the system of inequalities in Equation (6). To incorporate the used-defined cluster weights, *Q*_μ_, we modified the standard formulation of the SVM problem (Wang, 2005), and in this study maximize the following dual Lagrangian function in order to obtain the SVM weight vector ***w***:

(9) $$\begin{array}{l} L_{d}\left(\alpha \right)=\frac{1}{m}\sum_{i\;=\;1}^{l}\alpha_{i}-\frac{1}{2Nm^{2}}\sum_{i,j\;=\;1}^{l}\left({\left(\Delta x^{i}\right)}^{T}\Delta x^{j}\right)\alpha_{i}\alpha_{j}\\ 0\leq \alpha_{i}\leq CQ_{i},\;i=1,2,...,l\\ w=\frac{1}{m}\sum_{i\;=\;1}^{l}\alpha_{i}\Delta x^{i} \end{array}$$

In these expressions, *l* is the number of SVM support vectors and *C* is the SVM margin (see the inset in Figure 5B). Similar to the perceptron robustness, there is a large range of values of *C* for which the SVM produces reasonably good generalization results for both datasets. *C* = 2^20^ was used to produce results of this study. Again, all used-defined cluster weights, *Q*_μ_, were set to 1 during training.

### Active learning strategy

In this section we describe a pool-based sampling approach (Lewis and Gale, 1994) that can be used to actively train the Perceptron and SVM classifiers on branch merging examples. In this approach the user selectively draws queries from the pool of all branch merging clusters based on the value of the confidence measure:

(10) $$Confidence=\frac{e^{-w^{T}x^{correct\;merger}/T}}{\sum_{\begin{array}{c} i\in all\\ mergers \end{array}}e^{-w^{T}x^{i}/T}}$$

This measure assigns low confidence values (in the 0–1 range) to clusters in which the erroneous merging scenarios are located close to the decision boundary defined by ***w***. Parameter *T* controls the spread of confidence values but does not affect their order. This parameter was set to 1 throughout the study. Training can be performed after labeling a single or multiple low confidence clusters, and the confidence measure is updated after each training step. It is absolutely essential that clusters in which the correct merging scenario cannot be identified with high certainty should not be used for training, as a small number of errors in the labeled set may significantly worsen the performance of classifiers.

## Results

The methodology described in this study is implemented in the NCTracer software for automated tracing of neurites. This methodology consists of two major parts—initial tracing and branch merging. In the first part, an initial trace is created by using the Voxel Coding (Zhou et al., 1998; Zhou and Toga, 1999; Vasilkoski and Stepanyants, 2009) or the Fast Marching (Cohen et al., 1994; Cohen and Kimmel, 1997; Sethian, 1999; Mukherjee and Stepanyants, 2012) algorithm, and optimized to ensure that the trace, including its branch and terminal points, conforms well to the intensity in the underlying image (see the Methods section for details). Below we examine the initial traces from two very different dataset types: axons of single olfactory projection neurons from Drosophila (OP dataset, *n* = 9 image stacks) (Jefferis et al., 2007) and axons of multiple layer 6 neurons imaged in layer 1 of mouse visual cortex (L6 dataset, *n* = 6 image stacks) (De Paola et al., 2006). These datasets were featured at the DIADEM challenge (Brown et al., 2011) and serve as benchmarks for automated reconstruction algorithms. Figures 1A,B show representative image stacks from the OP and L6 datasets. The initial traces are superimposed on the maximum intensity projections of the image stacks, and are slightly shifted for better visibility. As can be seen, these initial traces accurately represent the geometry of neurites contained in the image stacks. However, a closer examination of the L6 trace topology reveals numerous erroneously merged (stolen) branches. Such errors in the initial trace often occur when the neurites belonging to different trees appear to be in contact due to poor *z*-resolution or due to high density of labeled structures. Presence of these topological errors becomes evident after labeling distinct tree structures with different colors (Figure 1C). The second part of our automated tracing algorithm uses a machine learning approach that actively learns the morphology of neurites in an attempt to resolve the errors present in the initial trace (see Figure 1D).

### Comparison of automated initial traces and manual user traces

**Figure 6:**
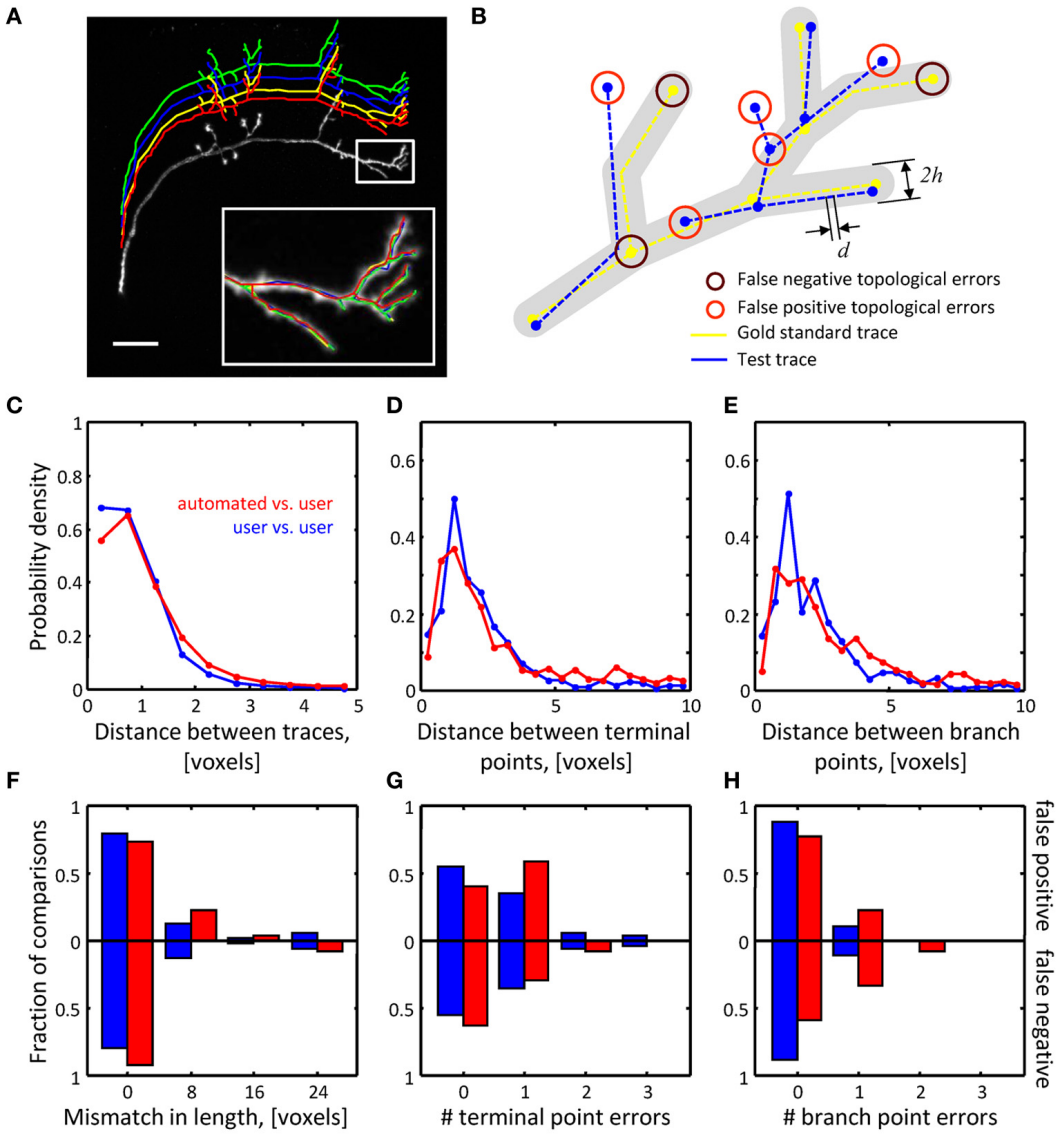
**Assessing the quality of automated traces. (A)** Three manual traces (green, blue, and yellow) and one automated trace (red) are superimposed on a maximum intensity projection of an OP neuron. The traces are staggered upward for better visibility. The inset shows a zoomed view of the boxed region. Scale bar is 20 μm. **(B)** Several geometrical and topological features are used to compare traces. Gold standard trace (yellow) and test trace (blue) are shown. Both traces are composed of nodes connected by edges of length *d*. Nodes on these traces are referred to as corresponding nodes if they are located within distance *h* of each other (*d* << *h*). Circles highlight false negative and false positive branch and terminal points. **(C–E)** Automated traces reliably capture the geometry of neurites. Nine OP axons were reconstructed with NCTracer, first automatically and then manually by three trained users. The probability densities for distances between the corresponding trace nodes **(C)**, terminal points **(D)**, and branch points **(E)** were used as metrics for geometrical comparisons. Red lines show the results of automated-to-user trace comparisons. Here, all user traces for every stack were used one by one as the gold standard, leading to 27 automated-to-gold standard trace comparisons. The results were pooled. Blue lines show similar results based on 54 user-to-user trace comparisons. **(F–H)** Automated traces accurately represent the topology of OP neurons. Three topological measures were compared: false positive/negative trace lengths **(F)**, numbers of false positive/negative terminal **(G)** and branch **(H)** points. Red and blue bars show the fractions of automated-to-user (*n* = 27) and user-to-user (*n* = 54) comparisons for different error types. The fractions for false positive and false negative errors are indicated with the bars above and below the *x*-axes.

Below we evaluate how well automated and manual traces capture the layout (geometry) of the neurites in the image stack, as well as how well they represent the morphology of branching tree structures (topology). Similar comparisons have been carried out in other studies (Gillette et al., 2011a; Choromanska et al., 2012; Mayerich et al., 2012). Each OP and L6 image stack was traced automatically using the Fast Marching algorithm as well as manually by three trained users. Figure 6A shows an example of the resulting four traces of a single OP stack. Inevitably, imperfect labeling and limited resolution of optical microscopy lead to uncertainties in tracing. Trained users often resolve such uncertainties differently from one another, and hence no single trace can be viewed as a gold standard. Thus, we had to first establish quantitative measures describing the baseline inter-user variability, and only then evaluate the performance of the automated tracing algorithm in comparison to this baseline. To this end, each manual trace was chosen to be the gold standard and compared to the automated trace and the remaining two manual traces. This led to 6 inter-user and 3 automated-to-user trace comparisons for each stack.

To ensure the uniformity of the reconstructed dataset, all traces were subdivided into segments of equal length (*d* = 0.25 voxels). To compare a pair of traces (a test trace and a gold standard trace) we perform a bi-directional nearest neighbor search to find corresponding nodes, i.e., nodes on the two traces separated by less than *h* = 10 voxels (see Figure 6B). A node in the test trace which has (does not have) a corresponding node in the gold standard trace is referred to as a true (false) positive node. A node in the gold standard trace for which there is no corresponding node in the test trace is referred to as a false negative node. Short terminal branches (less than 12 voxels) and dim branches (average intensity less than 0.12) were excluded from the comparisons.

**Figure 7:**
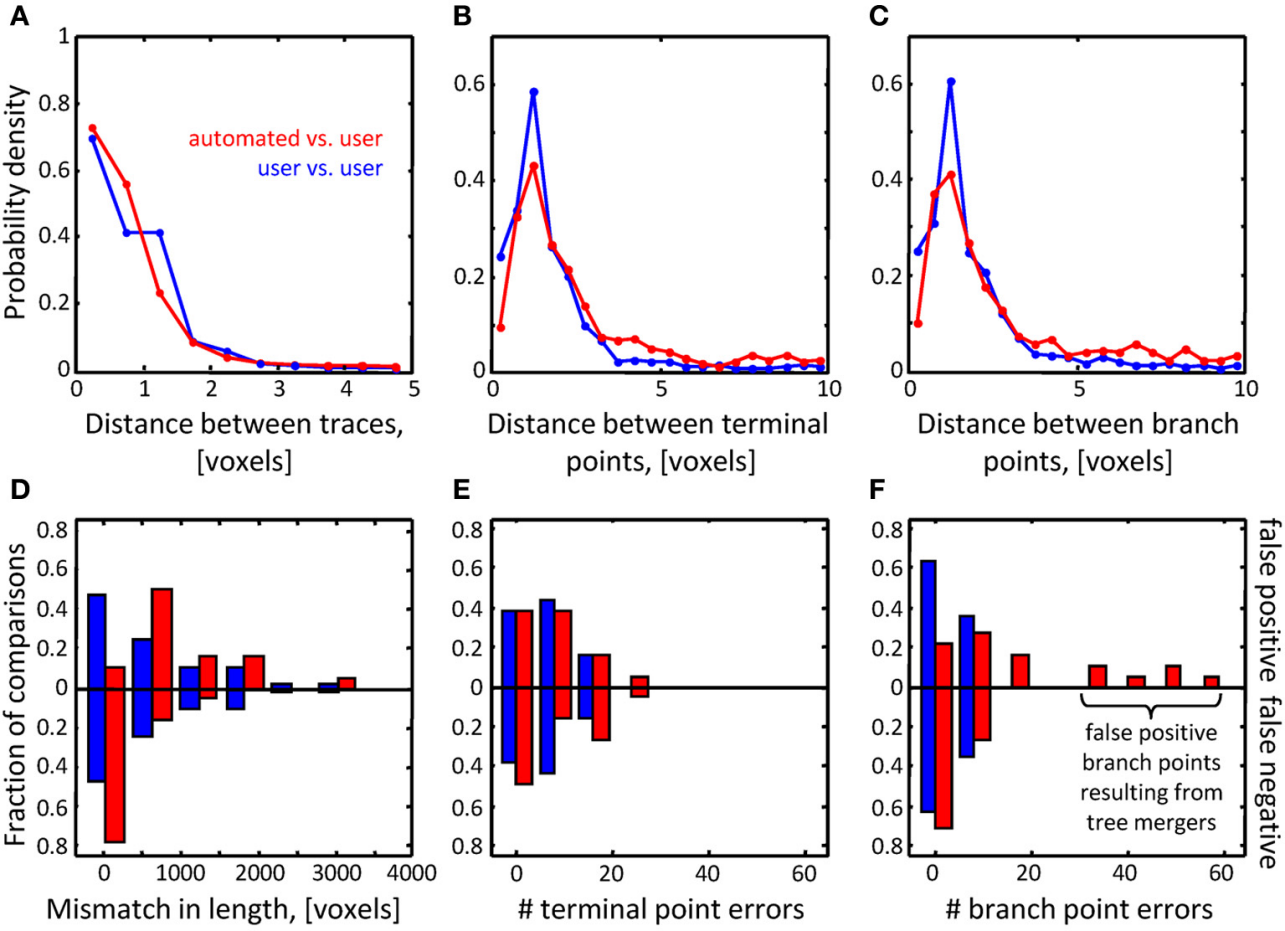
**Assessment of initial traces of multiple neuron axons**. Six L6 image stacks were reconstructed manually and automatically with NCTracer (see Figure 6 legend for details). **(A–C)** Automated traces reliably capture the geometry of neurites. The probability densities for distances between the corresponding trace nodes **(A)**, terminal points **(B)**, and branch points **(C)** were used as metrics for geometrical comparisons. Red lines (*n* = 18) and blue lines (*n* = 36) show the results of automated-to-user and user-to-user trace comparisons. **(D–F)** While the automated traces capture the geometry of the neurites well, they contain a markedly large number of false positive branch points **(F)**. These topological errors result from erroneous mergers of distinct axons that pass in close proximity of one another.

Results of the geometrical comparisons between automated initial traces and manual traces for the OP image stacks are shown in Figures 6C–E. The plots show probability densities of distances between corresponding nodes, corresponding branch points, and corresponding terminal points for both inter-user (blue lines), as well as automated-to-user comparisons (red lines). The geometrical precision of the automated and manual traces is evidenced by the fact that 95% of distance values lie below 2.3 voxels in Figure 6C, 7.3 voxels in Figure 6D, and 6.6 voxels in Figure 6E. More importantly, the difference between mean distances for the inter-user and automated-to-user comparisons (0.19, 0.51, and 0.65 voxels respectively) is smaller than the resolution of the image, and thus should have little bearing on trace dependent measurements. Similar conclusions were drawn from the geometrical comparisons of automated and manual traces of the L6 dataset (Figures 7A–C).

Topological errors that occur due to incorrectly merged branches are more difficult to detect and can be detrimental to circuit reconstruction projects. Three measures were selected to quantify the extent of such errors: false positive/negative trace lengths, numbers of false positive/negative terminal points, and numbers of false positive/negative branch points. The results of comparisons for the OP dataset (Figures 6F–H) show that similar numbers of topological errors were made by the algorithm and the users, and these numbers were generally small (less than one false positive/negative branch or terminal point per stack). For the L6 image stacks, the mismatches in length for the automated and manual traces were similar (Figure 7D), indicating that the automated algorithm performed as well as trained users in tracing the majority (in terms of length) of labeled structures. However, in contrast to manual traces, automated traces contained more false positive/negative terminal points (Figure 7E) and markedly larger number of false positive branch points (Figure 7F). The former errors result from branches that are broken due to imperfect labeling, while the latter arise from a specific artifact of the Fast Marching algorithm, i.e., merging nearby, but distinct branches. In particular, lower *z*-resolution of an image stack makes such mergers more prevalent, leading to larger numbers of false positive branch points.

### Active learning of branching morphology of neurites

To resolve the above mentioned topological errors, branches of the initial trace were disconnected from one another and merged into tree-like structures in an active learning approach described in the Methods section. Briefly, the positions of branch and terminal points were clustered based on distance, and branch merging was performed within every cluster independently (see Figure 4). Perceptron and SVM classifiers were designed and trained online to accomplish the branch merging task generating the final traces of the OP and L6 image stacks.

**Figure 8:**
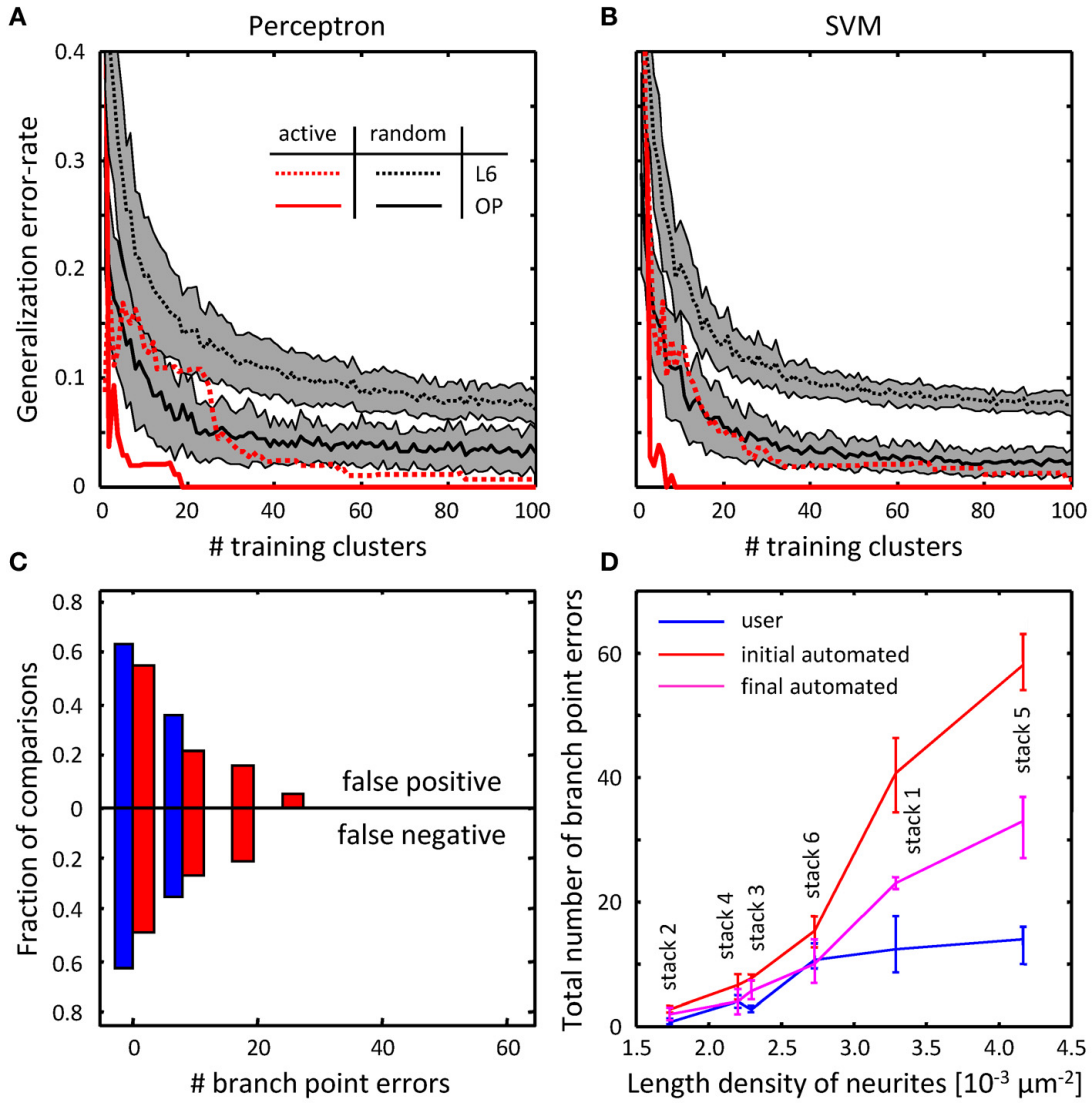
**Online training can be used to reduce the numbers of topological errors present in initial traces. (A)** Generalization error-rate as function of the number of training clusters for the perceptron classifier. Black lines (mean) surrounded by gray margins (standard errors) show the results of random training. For each number of training clusters, training sets were generated at random 1000 times. Training was performed on each set of clusters, while testing was done on the remaining clusters. Results for all 1000 experiments were averaged. Solid and dotted lines show the results for the OP and L6 datasets respectively. Red lines show the corresponding results for active training experiments. **(B)** Same for the SVM classifier. **(C)** The number of false positive branch points present in the initial trace of L6 dataset (Figure 7F) is greatly reduced by the branch merging algorithm. **(D)** The sum of false positive and false negative branch point errors is an increasing function of length density of labeled neurites. Length density is calculated as the average length of neurites traced by the users divided by the image stack volume. Error-bars indicate the range of branch point errors. Automated tracing algorithm performs as well as trained users when the density of labeled neurites is low (<0.003 μm^−2^).

To assess the performance of the classifiers, their generalization error rates were monitored as functions of the number of training examples. Figures 8A,B compare the performance of the classifiers trained on randomly selected branch merging examples with that of classifiers trained in an active learning approach. The plots show that the active learning approach provides a clear advantage in terms of the number of training examples required to reach a given error rate. For each dataset, error rates of less than 5% were achieved by both classifiers with less than 40 actively chosen training examples. The rapid decline of the generalization error rate validates our choice of features used for the branch merging task (see Figure 3). As expected, trained SVM and perceptron classifiers established nearly identical decision boundaries, as judged by the distance between their normalized weight-vectors (0.29 for OP and 0.10 for L6). In contrast, between-dataset distances were larger (0.63 for perceptron and 0.63 for SVM), indicative of the fact that classifiers were able to capture dataset specific morphological information.

Geometry and topology of the final automated traces produced by the branch merging algorithm were compared to the user traces in the manner described in Figures 6, 7. No significant geometrical changes resulted from automated branch merging. This was expected, as trace modifications that accompany branch merging are confined to very local regions in the vicinity of branch or terminal points. In addition, automated branch merging did not alter the topology of initial traces of OP neurites. The reason is that the initial traces of these morphologically simple structures did not contain significant topological errors in the first place (Figures 6F–H). As for the topology of L6 traces, no significant changes were observed in false positive/negative lengths (Figure 7D) and terminal point numbers (Figure 7E).

As was intended, automated branch merging greatly reduced the number of false positive branch points present in the initial traces (Figure 8C vs. Figure 7F). Though the reduction in the number of false positive branch points was large (about two-fold), the branch merging algorithm failed to achieve the level of user performance (Figure 8C). To examine the reason behind this disparity we plotted the sum of false positive and false negative branch point errors for every L6 stack as function of length density of neurites contained in the stack (Figure 8D). The length density is defined as the total length of traced neurites (in μm) divided by the stack volume (in μm^3^) and was calculated for each image stack by averaging over all user traces. These comparisons show that in every stack the branch merging algorithm substantially reduced the total number of errors present in the initial trace. What is more, when the density of labeled neurites was small (less than 0.003 μm^−2^, e.g., Figure 1D), the resulting final automated traces were on par with user reconstructions.

### Comparisons with other automated tracing tools

The geometrical and topological measures used to evaluate the quality of automated traces were also used to compare the performance of NCTracer, Vaa3D (Xiao and Peng, 2013), and NeuronStudio (Rodriguez et al., 2009). To this end, automated traces of OP and L6 image stacks were obtained with Vaa3D and NeuronStudio. We visually inspected these traces and varied the software parameters to achieve good coverage and performance. Inter-user and automated-to-user comparisons were performed as previously described. To evaluate the geometry of automated traces we calculated the mean distances between corresponding nodes and corresponding terminal and branch points. To assess the topology of automated traces, we obtained the Miss-Extra-Score (MES) for trace length and for the numbers of terminal and branch points (Xie et al., 2011). Trace MES is defined as the ratio of the gold standard length reduced by the false negative length to the gold standard length increased by the false positive length. Terminal and branch point MES are defined in a similar manner. The results of these comparisons are shown in Table 1.

**Table 1:** **Comparisons of manual and automated traces**.

	**OP dataset (single cell axons)**							**L6 dataset (axons of multiple cells)**						
	**Geometrical measures**			**Topological measures**			**Time**	**Geometrical measures**			**Topological measures**			**Time**
	**Trace**	**TP**	**BP**	**Trace**	**TP**	**BP**		**Trace**	**TP**	**BP**	**Trace**	**TP**	**BP**	
	**dist**.	**dist**.	**dist**.	**MES**	**MES**	**MES**		**dist**.	**dist**.	**dist**.	**MES**	**MES**	**MES**	
Inter-user	1.03 ± 0.02	2.18 ± 0.08	2.17 ± 0.09	0.995 ± 0.001	0.95 ± 0.01	0.98 ± 0.01	~15 min	1.01 ± 0.02	1.84 ± 0.05	1.80 ± 0.09	0.838 ± 0.023	0.90 ± 0.01	0.90 ± 0.01	~100 min
NCTracer	1.19 ± 0.02	2.57 ± 0.14	2.77 ± 0.08	0.996 ± 0.001	0.93 ± 0.01	0.94 ± 0.01	~20 s	1.07 ± 0.02	3.02 ± 0.07	3.13 ± 0.16	0.859 ± 0.024	0.86 ± 0.01	0.85 ± 0.02	~6 min
Vaa3D	1.39 ± 0.03	4.86 ± 0.13	3.46 ± 0.10	0.989 ± 0.002	0.77 ± 0.02	0.85 ± 0.02	~1 s	1.32 ± 0.01	6.04 ± 0.02	3.36 ± 0.10	0.736 ± 0.032	0.68 ± 0.02	0.68 ± 0.02	~25 s
NeuronStudio	2.19 ± 0.17	4.17 ± 0.24	4.19 ± 0.23	0.976 ± 0.006	0.85 ± 0.02	0.92 ± 0.02	~1 s	NA	NA	NA	NA	NA	NA	NA

*Manual traces were created by three trained users. Automated traces were generated with NCTracer as described in this study, as well as Vaa3D (Xiao and Peng, 2013) and NeuronStudio (Rodriguez et al., 2009) automated tracing software. Parameters for Vaa3D and NeuronStudio software were tuned to the best of our knowledge. Because NeuronStudio does not automatically trace neurites of multiple cells, this software was not used for the L6 dataset (NA's in the table). Geometrical and topological measures used are described in the text. Their numerical values are given in mean ± s.e.m format (n = 9 for OP and n = 6 for L6). All calculations were performed on a 3.59 GHz, 24 GB workstation running Windows 7. The following abbreviations are used in the table: dist., distance; TP, terminal points; BP, branch points; MES, miss-extra-score (Xie et al., 2011)*.

Smaller distance and higher MES indicate greater affinity between test and gold standard traces. Table 1 shows that all automated tracing tools were able to capture trace geometry and topology of single OP axons reasonably well. The advantage of the branch merging strategy proposed in this study becomes evident from examining the values of topological measures for L6 stacks, which contain multiple axons. According to these measures, NCTracer significantly outperforms other software. And in general, all geometrical and topological measures of NCTracer are closest to the inter-user measures. Table 1 also shows a trade-off between the quality of automated traces and tracing time. Vaa3D and NeuronStudio are 15–20 fold faster than NCTracer. We do not view this as a major drawback because tracing of single stacks with the current version of NCTracer can be easily performed on modern day workstations, while high-throughput projects could still be carried out on computer clusters.

## Discussion

Much of our understanding of brain structure and function is derived from quantitative analyses of neuron shapes. Researchers routinely utilize partial or complete single cell reconstructions, as well as reconstructions of multiple cells often spanning several stacks of images in order to address various questions. Single cell reconstructions are often used in cell classification and comparative neuroanatomy studies, theoretical studies of neuron shapes, and detailed computational models of intracellular activity. Single cell reconstructions are frequently pooled *in silico* to simulate structural connectivity of local neural circuits. Reconstructions of multiple labeled cells are used for the analyses of synaptic connectivity in local circuits, *in vivo* studies of circuit plasticity, and large-scale brain mapping projects. There is no doubt that automating the tracing process will advance these studies, significantly increasing their throughput and eliminating the biases and variability associated with manual tracing.

It is important to understand that it is usually not sufficient to obtain the basic layout of all labeled neurites. In particular, projects aimed at the analyses of synaptic connectivity require accurate knowledge of branching morphology of individual cells (Figure 1). In this study, we use machine learning to evaluate topologically different scenarios of constructing automated traces (Figure 4A) and then determine the correct branching pattern based on previously learned morphological features. A machine learning approach to image processing typically requires a large labeled set of examples, and creating such a set can be very time-consuming. Our active learning strategy circumvents this problem by taking advantage of the combinatorial nature of the numbers of branch merging scenarios (Figure 4B). Another advantage of this strategy is subtractive normalization, Equation (6). Branch merging scenarios are only compared within clusters, normalizing for the variations in local intensity and density of labeled neurites.

The results of this study show that the quality of automated traces is strongly dependent on the length density of labeled neurites. When this density is lower than 0.003 μm^−2^ the automated tracing algorithm performs on par with trained users (Figure 8D); the reliability of automated traces diminishes rapidly with increase in density beyond this point. Hence, proofreading and error-correction may be required for some automated traces. Proofreading must be done in a computer guided manner, which is particularly important for high-throughput reconstruction projects. The confidence measure described in Equation (10) can be used to convey information about the certainty in the outcome of automated tracing. This measure can be calculated for every vertex in the trace and can be used to direct the user's attention to the most uncertain parts of the trace. Only the lowest confidence mergers will need to be examined by the user, leading to a substantial reduction in proofreading time. Such low confidence regions can be highlighted automatically and the user would choose from an ordered set of best alternative scenarios (based on decreasing confidence).

With the automation of tracing and proofreading it should be possible to map intact, sparsely labeled circuits on the scale of a whole brain, e.g. in the fly or the mouse. Consider a hypothetical experiment of mapping structural connectivity in the mouse brain. The adult mouse brain is roughly 500 mm^3^ in volume (Ma et al., 2005). Subsets of mouse neurons can be labeled *in vivo* to reveal the layout of their axonal and dendritic arbors. The brain can then be divided into 0.5 × 0.5 × 0.1 mm^3^ optical sections, and imaged in 3D with two-photon or confocal microscopy at 0.5 × 0.5 × 1.0 μm^3^ spatial resolution. This procedure would result in 20,000 stacks of images, each composed of 1000 × 1000 × 100 voxels, totaling 2 TB of raw imaging data. A dataset of this size would have to be reconstructed on a high-performance computer cluster, and the results could be viewed and proofread on modern-day workstations. Depending on the density of labeling, reconstruction of a single stack may take on the order of 1 core-hour, or 20,000 core-hours for the entire brain. Thus, whole mouse brain mapping is no longer an unfeasible goal.

Brain mapping at a much lower spatial resolution has long been performed with diffusion tensor imaging (DTI). This non-invasive technique measures the diffusion tensor associated with anisotropic movement of water molecules along white matter fiber bundles. Numerous algorithms have been developed to construct tracts from such information, establishing coarse-grain connectivity between brain regions (for review see Le Bihan, 2003; Hasan et al., 2011; Soares et al., 2013). Such algorithms typically use streamline tractography to connect voxels of similar tensor-field orientations into a trace. The reconstruction problem encountered in DTI is somewhat related to the problem of neurite tracing described in this study; however, there is an important difference. Due to the relatively low resolution of DTI (typically 1 mm^3^ per voxel) there is no anatomical basis for trying to detect branching patterns in DTI tracts. Hence, unlike neurite tracing, where topological errors may have a catastrophic effect on the overall connectivity map, the results of DTI tracing are expected to be less sensitive to such errors. It remains to be seen to what extent brain mapping at single neuron resolution correlates with the connectivity maps established with DTI.

### Conflict of interest statement

The authors declare that the research was conducted in the absence of any commercial or financial relationships that could be construed as a potential conflict of interest.
